# Personal

**Published:** 1908-02

**Authors:** 


### PERSONAL.

Dr. E. Gustav Zinke, of Cincinnati, President of the American Association of Obstetricians and Gynecologists, was the guest of the Wayne County Medical Society at Detroit, Monday, January 20, 1908. In the evening he read a paper before the society entitled, "Is there room for improvement in the practice of obstetrics?"

Dr. Charles F. Stokes, surgeon United States Navy, has been directed to assume the command of the Hospital Ship Relief, now fitting out at the navy yard, Mare Island, Cal., for special duty with the battleship fleet at present on the way to Pacific waters.

Dr. E. L. Keyes, Jr., of New York, will read a paper before the Buffalo Academy of Medicine on February 10, 1908. Dr. Keyes is one of the best known of the younger genitourinary surgeons in New York, and his recently published work on syphilis, based on his own clinical work has placed him in the first rank of authors on syphilography.

Dr. Herbert L. Burrell, of Boston, president-elect of the American Medical Association, has been appointed John Homans professor of surgery at the Harvard Medical School.

Dr. H. H. Bingham, of Buffalo, has been elected president of the Board of Councilmen. Dr. Bingham's previous service in the board would seem to entitle him to this distinction.

